# Postoperative lateral-wall status and fixation failure after cephalomedullary nailing of extracapsular hip fractures: an ambispective cohort study

**DOI:** 10.3389/fmed.2026.1922104

**Published:** 2026-10-28

**Authors:** Mengyu Wang, Chenxiao Zuo, Ze Zhang, Fengpo Sun, Liangyuan Wen

**Affiliations:** 1 Department of Orthopedics, Beijing Hospital, National Center of Gerontology, Institute of Geriatric Medicine, Chinese Academy of Medical Sciences, Beijing, China; 2 Chinese Academy of Medical Sciences & Peking Union Medical College, Beijing, China; 3 School of Medicine, Shandong Xiandai University, Jinan, Shandong, China

**Keywords:** cephalomedullary nailing, extracapsular hip fracture, fixation failure, postoperative lateral-wall status, prognosis

## Abstract

**Background:**

Extracapsular hip fractures cause substantial morbidity in older adults, and cephalomedullary nailing is widely used for fixation. Although lateral-wall disruption is an established concern in extramedullary fixation, the prognostic relevance of postoperative lateral-wall status after cephalomedullary nailing remains uncertain. We evaluated its association with fixation failure among patients who achieved satisfactory reduction.

**Methods:**

An ambispective cohort of patients with acute extracapsular hip fractures treated with cephalomedullary nailing at Beijing Hospital (January 2010–October 2025) was analyzed. Only patients who achieved excellent or acceptable reduction were included. Postoperative lateral-wall status was classified using preoperative lateral-wall thickness and fracture morphology together with any new intraoperative lateral-wall fracture identified on postoperative imaging. Radiographic fixation failure was the primary endpoint; follow-up was at least 6 months unless failure was confirmed earlier. Failure-free survival and the association between postoperative lateral-wall status and fixation failure were evaluated using Kaplan–Meier analysis and prespecified parsimonious Firth penalized logistic regression models.

**Results:**

Among 508 patients, 33 (6.5%) experienced fixation failure: 16 of 249 (6.4%) with complete and 17 of 259 (6.6%) with incomplete postoperative lateral-wall status. The log-rank test yielded *p* = 0.778. In the Firth model adjusted for age and tip–apex distance, no statistically significant association was detected between postoperative lateral-wall status and fixation failure (incomplete vs. complete: adjusted odds ratio, 1.04; 95% CI: 0.52–2.10; *p* = 0.915).

**Conclusion:**

Among patients with AO/OTA 31-A extracapsular hip fractures who achieved satisfactory reduction, we did not detect a statistically significant association between postoperative lateral-wall status and fixation failure after cephalomedullary nailing. Because the analysis was restricted to satisfactorily reduced fractures and the number of failure events was limited, these findings should not be interpreted as evidence that lateral-wall morphology is unimportant in the broader fracture population.

## 1 Introduction

Acute AO/OTA 31-A extracapsular hip fractures are common osteoporotic injuries in older adults, usually resulting from low-energy falls and occurring more frequently in women (1, 2). They are associated with substantial mortality and limited functional recovery, particularly among older patients and those with major comorbidities (3–6). With global population aging, their incidence and associated economic burden are expected to rise, making these fractures an important public health concern (7–9).

Conservative management of extracapsular hip fractures is associated with unfavorable clinical outcomes. In observational geriatric hip-fracture cohorts that included extracapsular hip fractures, 1-year mortality was approximately two- to threefold higher after nonoperative than operative treatment (10–12). Consequently, surgical intervention remains the preferred treatment strategy for extracapsular hip fractures, provided the patient's condition allows for operative management (13).

Historically, the management of extracapsular hip fractures relied primarily on extramedullary fixation techniques, including plate-screw constructs and dynamic hip screws (DHS). In 2004, Gotfried introduced the concept of the femoral trochanteric lateral wall (14). From a biomechanical perspective, the medial proximal femur bears compressive stress, whereas the lateral wall is subjected to tensile stress. In extramedullary fixation, the absence of the lateral wall eliminates essential support against tensile stress, predisposing the proximal femur to hip varus, implant cut-out, and fixation failure, thereby substantially elevating the risk of mechanical failure (14, 15). Revision surgery is often necessitated by fixation failure, further exacerbating patient morbidity. More recently, lateral wall fixation plates have been introduced to improve tensile strength in proximal femoral fractures involving the lateral wall. However, placement of these plates necessitates additional dissection and exposure, leading to increased blood loss and prolonged operative time (16). Hsu et al. defined lateral-wall thickness and reported that a preoperative thickness <20.5 mm was associated with an increased risk of postoperative lateral-wall fracture after sliding hip-screw fixation (17). In the present study, this threshold was used together with preoperative fracture morphology and any new intraoperative lateral-wall fracture identified on immediate postoperative imaging to classify postoperative lateral-wall status as complete or incomplete.

Cephalomedullary nailing creates a biomechanical environment distinct from that of sliding hip-screw fixation because its intramedullary position shortens the lever arm and may provide an internal lateral buttress (18, 19). Evidence concerning the lateral wall in extramedullary fixation therefore cannot be directly extrapolated to intramedullary constructs. Although lateral-wall disruption has been implicated in fixation failure after cephalomedullary nailing (20, 21), the prognostic relevance of postoperative lateral-wall status remains uncertain.

Incomplete lateral-wall morphology may reflect greater fracture complexity and make satisfactory reduction more difficult. Prior clinical and biomechanical work suggests that reduction quality, medial cortical support, and cortical apposition are relevant to construct stability (16, 22, 23), while the concept of positive anteromedial cortical support (PACS) emphasizes anteromedial cortical contact as a potential pathway for load transfer (24, 25). However, medial cortical support, PACS, and anteromedial cortical contact were not assessed as separate variables in this cohort. Accordingly, this study evaluated whether postoperative lateral-wall status was associated with fixation failure after cephalomedullary nailing among patients with extracapsular hip fractures who achieved satisfactory reduction.

## 2 Methods

### 2.1 Study design

This study was approved by the Ethics Committee of Beijing Hospital (Approval No. 2023BJYYEC-290-01). It was registered in the National Medical Research Registration and Recordation System (Record No. MR-11-23-037970) and the Chinese Clinical Trial Registry (Registration No. ChiCTR2500096614). We conducted a single-center ambispective cohort study of consecutive patients with extracapsular hip fractures treated with cephalomedullary nailing at Beijing Hospital. Eligible patients were retrospectively identified between January 2010 and June 2023 and prospectively enrolled between July 2023 and October 2025. Written informed consent was obtained from all prospectively enrolled patients, whereas the requirement for informed consent was waived for the retrospective cohort. The cephalomedullary devices used were the Proximal Femoral Nail Antirotation (PFNA), Gamma 3, and InterTAN systems. Throughout the study period, treatment followed the same core operative principles and a criteria-based postoperative weight-bearing strategy, although implant selection evolved over time. For study assessments, all available preoperative, immediate postoperative, and follow-up images were reassessed using the same prespecified definitions. Detailed treatment, participant screening, imaging assessment, and data quality-control procedures are provided in the Supplementary Methods. The study was conducted in accordance with applicable institutional and national ethical requirements and the Declaration of Helsinki (2013 revision). Because the analytic cohort was restricted to patients who achieved satisfactory postoperative reduction, the primary analysis addressed the association between postoperative lateral-wall status and fixation failure within this selected population rather than the overall prognostic association of preoperative fracture morphology among all patients undergoing cephalomedullary nailing.

### 2.2 Participants

During the retrospective study period, all consecutive patients who underwent cephalomedullary nailing were identified from the institutional surgical database and screened for eligibility. During the prospective study period, consecutive patients were assessed using the same clinical and imaging eligibility criteria, with final eligibility confirmed after postoperative assessment of reduction quality. Participant selection was reported in accordance with the STROBE statement and, for the retrospective database component, the RECORD extension (26, 27).

Patients were eligible if they (1) had an acute extracapsular hip fracture (28), fulfilled routine indications for operative treatment, and underwent cephalomedullary nailing; and (2) achieved excellent or acceptable reduction (hereafter termed satisfactory reduction) on immediate postoperative anteroposterior and lateral radiographs according to the Baumgaertner reduction quality criteria (29). Exclusion criteria were (1) multiple injuries; (2) surgery more than 3 weeks after injury; (3) pathological fracture; or (4) a preoperative condition precluding surgery. Eligibility for the final analysis required at least 6 months of radiographic follow-up, unless fixation failure was confirmed earlier. Before exclusion for insufficient follow-up in either cohort phase, all available postoperative records and imaging studies through the last documented contact were reviewed to determine whether fixation failure had already occurred. Patients with fixation failure confirmed within 6 months were retained irrespective of subsequent follow-up duration because the primary endpoint and event date had already been ascertained. Initial sample-size planning was based on anticipated fixation-failure proportions according to postoperative lateral-wall status; the assumptions, calculation method, and scope of this planning calculation are detailed in the Supplementary Methods.

### 2.3 Measurements

At baseline, demographic, clinical, and imaging data were collected, including sex, age, body mass index (BMI), American Society of Anesthesiologists (ASA) classification, relevant medical history, perioperative information, preoperative and postoperative day-1 anteroposterior and lateral radiographs, and preoperative three-dimensional CT scans when available. Extracapsular hip fractures were classified according to the 2018 AO/OTA criteria (28).

Preoperative anteroposterior radiographs obtained under traction with the limb in neutral rotation were used to measure lateral-wall thickness according to the method described by Hsu et al. (17) (Figure 1). Preoperative three-dimensional CT scans were additionally reviewed to characterize fracture morphology and identify coronal lateral-wall fractures that were not clearly visible on plain radiographs. Postoperative day-1 radiographs were compared with the preoperative images to identify new lateral-wall fractures occurring intraoperatively.

**Figure 1:**
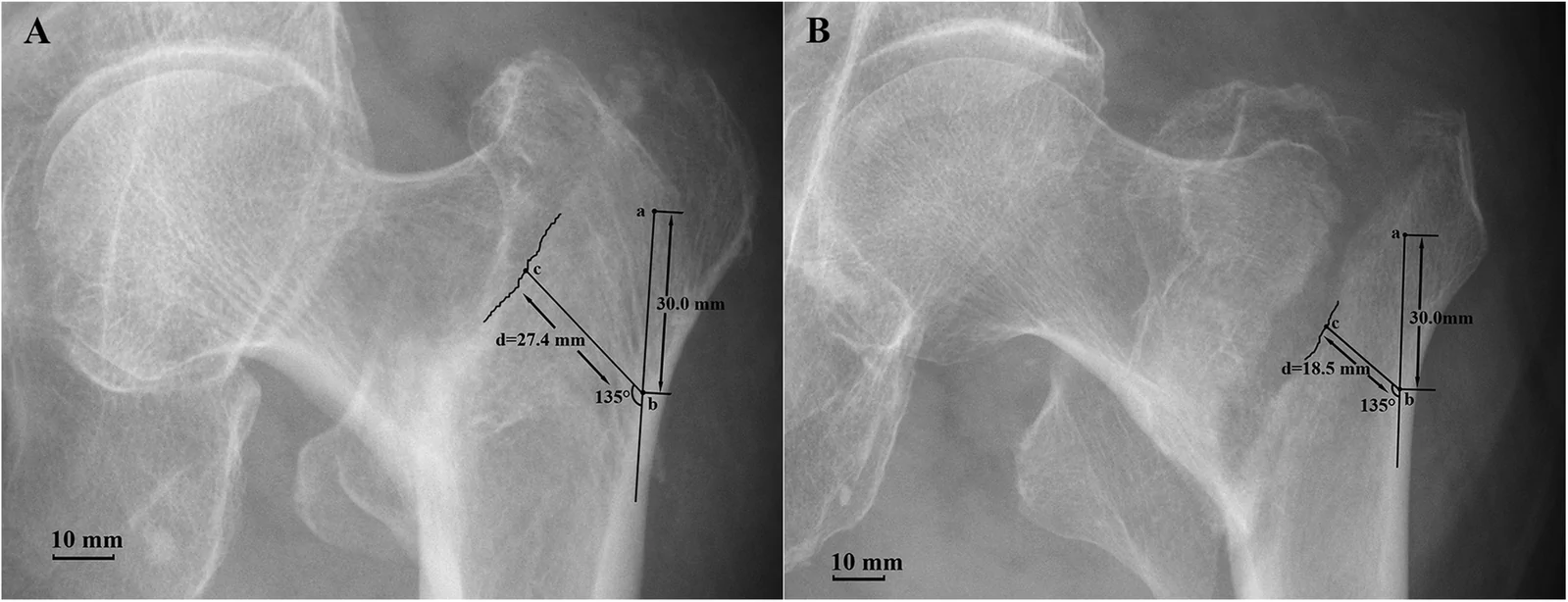
Preoperative radiographic measurement of lateral-wall thickness. On an anteroposterior radiograph obtained under traction with the limb in neutral rotation, point b was identified 3 cm distal to the innominate tubercle of the greater trochanter (point a) along the femoral shaft. From point b, a line was drawn proximally at an angle of 135° to the femoral shaft until it intersected the fracture line at point c. The linear distance (d) from point b to point c along this line was recorded as the lateral-wall thickness (17). **(A,B)** show measurements ≥20.5 mm and <20.5 mm, respectively. This preoperative thickness measurement was one component of the postoperative lateral-wall status classification and was not itself the study exposure.

Postoperative lateral-wall status was classified from preoperative fracture morphology and postoperative imaging. For AO/OTA 31-A1 and 31-A2 fractures, the lateral wall was classified as incomplete when the preoperative thickness was <20.5 mm, a coronal lateral-wall fracture was present, or a new intraoperative lateral-wall fracture was identified; otherwise, it was classified as complete. AO/OTA 31-A3 fractures were classified as having an incomplete lateral wall because the fracture-line morphology involved the lateral cortex; the 20.5 mm thickness threshold was not applied to these fractures.

Reduction quality was assessed on postoperative radiographs according to the Baumgaertner–Solberg criteria (29), and tip–apex distance (TAD) was measured on anteroposterior and lateral radiographs after implant-specific magnification correction (Supplementary Figures S1, S2). Anteromedial cortical support was not recorded as a standalone variable and was not used for group allocation or regression modeling.

Patients without earlier confirmed fixation failure underwent radiographic follow-up for at least 6 months, and all subsequent assessments were retained through the last available follow-up. At each documented visit, anteroposterior and lateral radiographs of the affected hip were reviewed; three-dimensional CT images were additionally reviewed when obtained for clinical indications. For patients with fixation failure, follow-up time was calculated from surgery to the first imaging examination confirming the event. Patients without fixation failure were censored at the last imaging examination confirming its absence.

Fixation failure was defined as a composite radiographic endpoint comprising penetration, cut-out, backing-out, main-nail breakage, nonunion, and varus collapse; individual components could coexist. Penetration was defined as medial intra-articular migration of the cephalic fixation element, cut-out as its superior or anterosuperior extrusion with varus collapse, and backing-out as abnormal lateral withdrawal beyond controlled telescoping. Main-nail breakage was defined as radiographic discontinuity of the nail, and nonunion as a persistent fracture line without progressive bridging callus at 6 months or later. Varus collapse was defined as a decrease in the neck-shaft angle to <110° relative to the immediate postoperative alignment. No additional angular-change threshold or confirmation on consecutive examinations was required, and the first image meeting the criterion defined the event date. Penetration secondary to femoral head necrosis was excluded.

All imaging variables and fixation outcomes were independently assessed by two trained orthopedic reviewers, with disagreements adjudicated by a third senior orthopedic trauma surgeon; blinding and other quality-control procedures are detailed in the Supplementary Methods.

### 2.4 Statistical analysis

Continuous variables were presented as mean ± standard deviation (SD) or median (interquartile range) and compared between groups using appropriate parametric or nonparametric tests. Categorical variables were summarized as frequencies and percentages and compared using the chi-square or Fisher's exact test. Missingness was evaluated using Little's test of missing completely at random (MCAR). Missing covariate data were handled using multiple imputation by chained equations with predictive mean matching; 20 imputed datasets were generated, and estimates from analyses involving imputed variables were combined using Rubin's rules. Failure-free survival was estimated using Kaplan–Meier analysis and compared with the log-rank test; patients without fixation failure were censored at the last radiographic examination confirming the absence of failure. Cumulative failure rates at prespecified time points, Cox proportional hazards models, and restricted mean survival time (RMST) analysis at 24 months were used as supplementary time-to-event analyses.

To identify factors associated with fixation failure, univariable and multivariable Firth penalized logistic regression models were fitted to reduce small-sample bias. Given the limited number of fixation-failure events relative to the candidate predictors, a prespecified parsimonious modeling strategy was used rather than a single fully adjusted saturated model. Postoperative lateral-wall status was the main exposure, and TAD was retained as a core technical covariate. Additional covariates were entered one at a time in separate models: age (Model A), AO/OTA classification (Model B), reduction quality (Model C), ASA grade (Model D), and implant type (Model E). One *post hoc* sensitivity analysis additionally incorporated cohort phase (retrospective vs. prospective) into the core model to explore potential temporal confounding. A second *post hoc* sensitivity analysis excluded AO/OTA 31-A3 fractures and refitted the core model to assess whether their morphology-based assignment to the incomplete category influenced the primary estimate. All prespecified subgroup and interaction analyses were considered exploratory because of the limited number of events within strata. Given the resulting limitations in statistical power and precision, multivariable and exploratory estimates were interpreted with emphasis on point estimates and 95% CIs; nonsignificant findings were not considered evidence of no association. All statistical tests were two-sided, and *p* < 0.05 was considered statistically significant. Analyses were performed using IBM SPSS Statistics version 25 and R version 4.4.1. Additional statistical details are provided in the Supplementary Methods.

## 3 Results

From January 2010 to June 2023, 1,420 patients who underwent cephalomedullary nailing were screened for the retrospective cohort. Of these, 1,007 had less than 6 months of radiographic follow-up and no radiographically confirmed fixation failure during their available follow-up and were therefore excluded. Among the remaining 413 patients, 48 were excluded because surgery was performed more than 3 weeks after injury (*n* = 8), pathological fracture was present (*n* = 1), multiple injuries were present (*n* = 27), medical records were incomplete (*n* = 1), previous surgery had been performed for the index fracture (*n* = 2), the fracture did not meet the definition of an acute AO/OTA 31-A extracapsular hip fracture (*n* = 7), perioperative data were incomplete (*n* = 1), or reduction quality was poor (*n* = 1), leaving 365 patients in the retrospective cohort. From July 2023 to October 2025, 205 patients underwent cephalomedullary nailing; 16 were excluded because of pathological fractures (*n* = 2), multiple injuries (*n* = 4), incomplete perioperative data (*n* = 1), surgery more than 3 weeks after injury (*n* = 2), poor reduction quality (*n* = 4), or fractures not meeting the definition of an acute AO/OTA 31-A extracapsular hip fracture (*n* = 3). Of the 189 eligible prospective patients, 46 had not completed the minimum 6-month follow-up at the time of analysis and had no radiographically confirmed fixation failure during their available follow-up, leaving 143 patients in the prospective cohort. Review of the last available records and images confirmed that none of the patients excluded for insufficient follow-up in either cohort had radiographically confirmed fixation failure during their available follow-up. In total, 508 patients were included in the final analysis (Figure 2).

**Figure 2:**
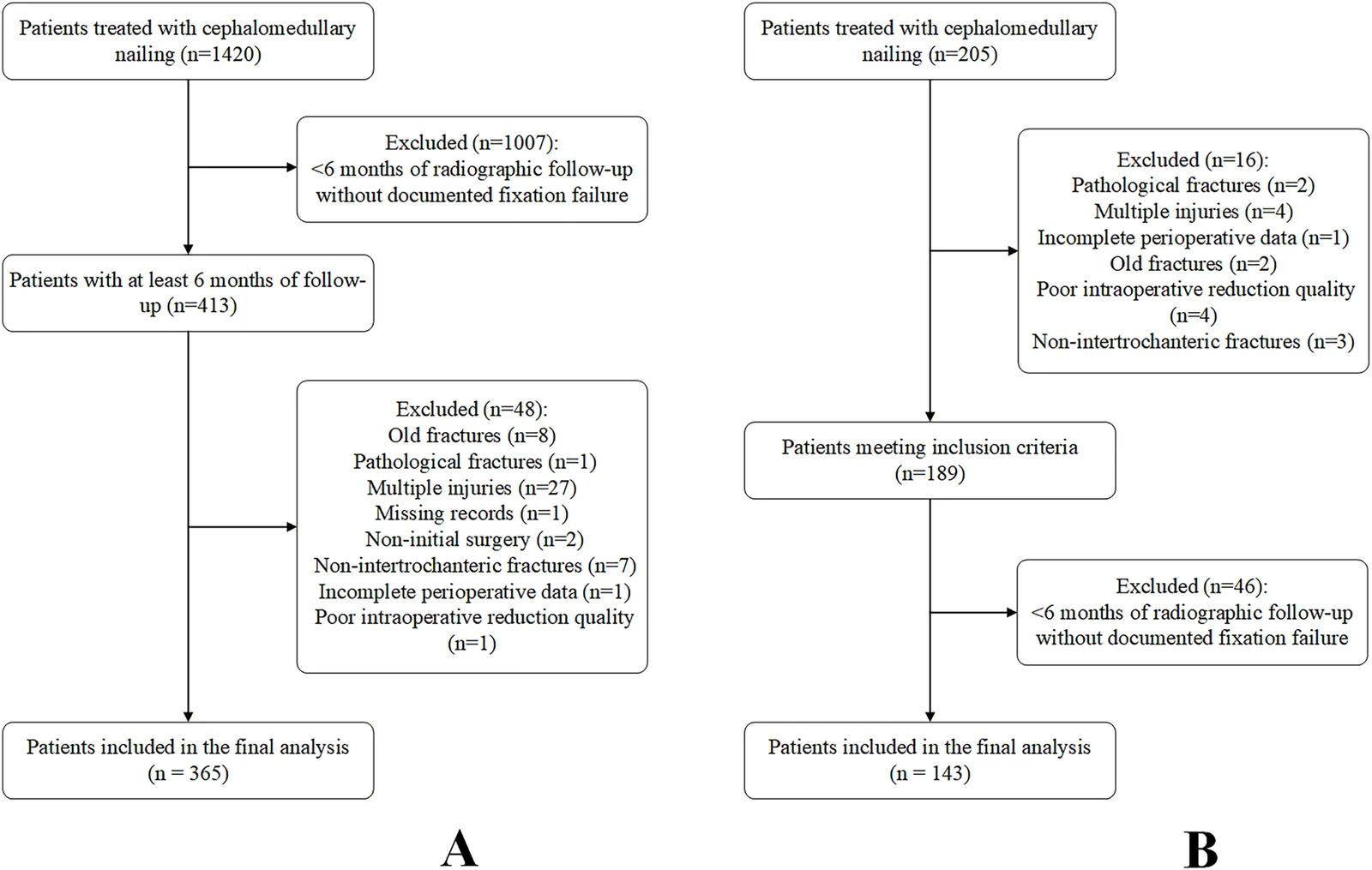
The detailed patient selection process in this study: **(A)** depicts the patient selection process for the retrospective cohort, whereas **(B)** illustrates the selection process for the prospective cohort.

Missing values occurred in 31 patients (6.1%): BMI was missing in all 31, whereas ASA grade and five perioperative variables were additionally missing in one of these patients. Little's MCAR test did not provide evidence against the MCAR assumption (*χ*^2^ = 35.11, df = 28, *p* = 0.167). Twenty imputed datasets were generated, and convergence diagnostics showed no material concerns.

During follow-up, 33 patients (6.5%) experienced fixation failure. The seven observed radiographic patterns were isolated penetration of the cephalic fixation element (*n* = 8), isolated varus collapse (*n* = 4), penetration with varus collapse (*n* = 9), main-nail breakage with varus collapse (*n* = 2), penetration with backing-out (*n* = 2), varus collapse with backing-out (*n* = 5), and penetration with both backing-out and varus collapse (*n* = 3). One additional case of penetration secondary to postoperative femoral head necrosis was excluded from the primary composite endpoint. Representative patterns are shown in Figure 3.

**Figure 3:**
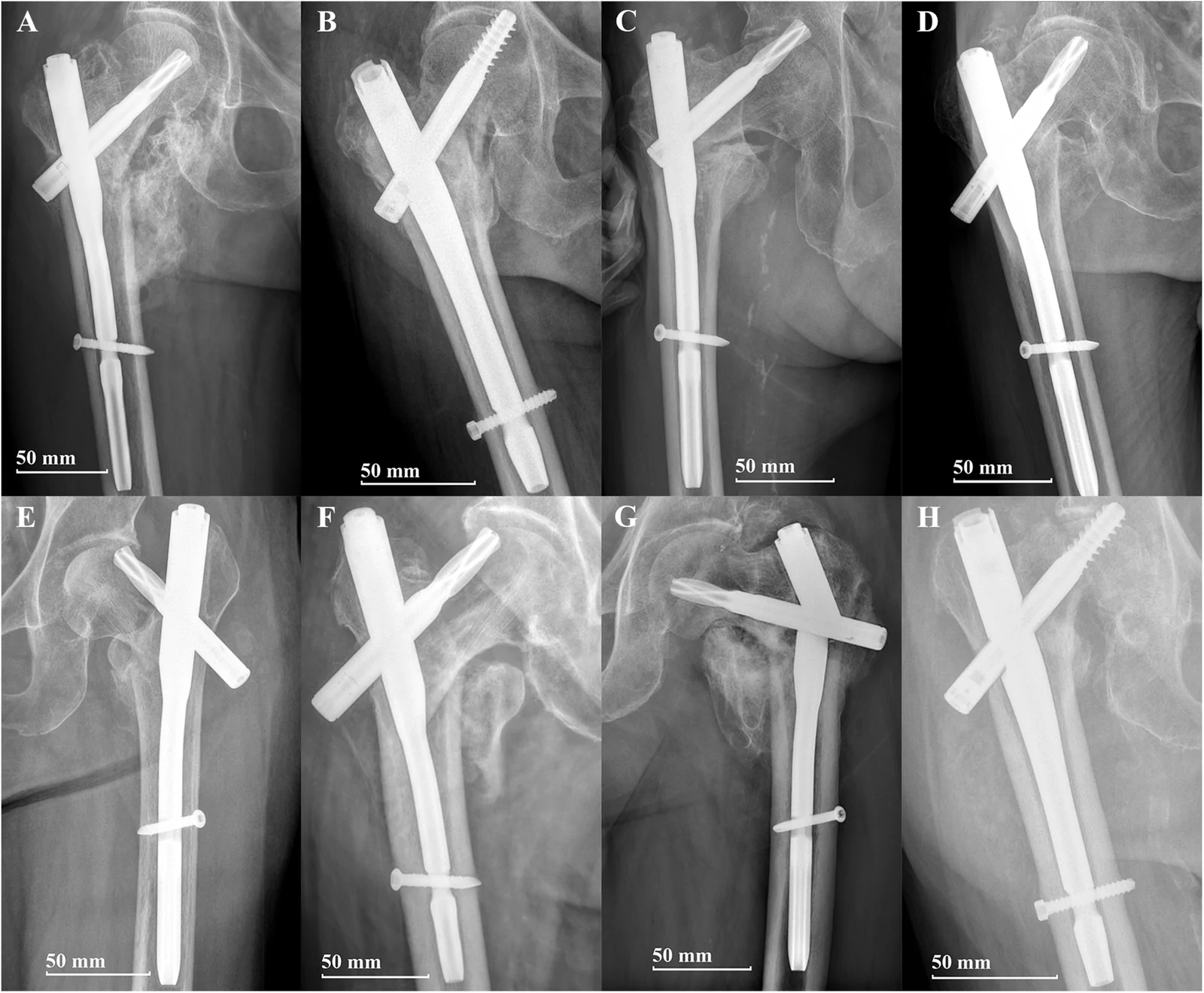
Representative radiographic patterns of fixation failure and an excluded penetration case. **(A)** Isolated varus collapse. **(B)** Isolated penetration of the cephalic fixation element. **(C)** Penetration with varus collapse. **(D)** Varus collapse with backing-out. **(E)** Penetration with backing-out. **(F)** Penetration with backing-out and varus collapse. **(G)** Main-nail breakage with varus collapse. **(H)** Penetration secondary to postoperative femoral head necrosis. Panels **A–G** represent events included in the primary composite endpoint. Panel **H** was excluded because the penetration was attributed to femoral head necrosis rather than mechanical failure of the fracture–implant construct.

### 3.1 Baseline characteristics and group comparison

A total of 508 patients were analyzed: 249 (49.0%) were classified as having complete and 259 (51.0%) as having incomplete postoperative lateral-wall status. The groups were comparable in age (79.31 ± 10.21 vs. 79.48 ± 10.45 years, *p* = 0.537), BMI (23.02 ± 3.70 vs. 23.16 ± 3.62 kg/m^2^, *p* = 0.449), sex distribution (67.9% vs. 71.4% female, *p* = 0.383), ASA grade (*p* = 0.226), fracture side (*p* = 0.849), implant type (*p* = 0.260), TAD (22.59 ± 6.53 vs. 23.33 ± 6.61 mm, *p* = 0.166), and follow-up time (309.76 ± 209.01 vs. 285.88 ± 132.00 days, *p* = 0.454). The incomplete group had longer operation times (78.95 ± 28.06 vs. 72.37 ± 28.44 min, *p* < 0.001), greater intraoperative blood loss (205.75 ± 74.26 vs. 188.35 ± 65.57 mL, *p* < 0.001), greater intraoperative transfusion volumes (259.54 ± 156.94 vs. 210.72 ± 175.39 mL, *p* = 0.001), and a lower rate of excellent reduction (66.4% vs. 78.7%, *p* = 0.002). Other parameters did not differ significantly (Table 1).

**Table 1:** Demographic, perioperative, and radiographic characteristics according to postoperative lateral-wall status.

Variable	Total *n* = 508	Complete *n* = 249	Incomplete *n* = 259	Statistic (z/*χ*^2^)	*p*
Age (years)	79.40 ± 10.32	79.31 ± 10.21	79.48 ± 10.45	−0.617	0.537
BMI (kg/m^2^)	23.10 ± 3.66	23.02 ± 3.70	23.16 ± 3.62	−0.757	0.449
Sex				0.76	0.383
Male	154 (30.3%)	80 (32.1%)	74 (28.6%)		
Female	354 (69.7%)	169 (67.9%)	185 (71.4%)		
ASA grade				−1.211	0.226
I	11 (2.2%)	7 (2.8%)	4 (1.5%)		
II	272 (53.5%)	138 (55.4%)	134 (51.7%)		
III	217 (42.7%)	100 (40.2%)	117 (45.2%)		
IV	8 (1.6%)	4 (1.6%)	4 (1.5%)		
Time from injury to operation (days)	6.24 ± 3.64	6.22 ± 3.75	6.25 ± 3.54	−0.266	0.790
Operation time (minutes)	75.73 ± 28.41	72.37 ± 28.44	78.95 ± 28.06	−3.614	**<0**.**001**
Intraoperative blood loss (mL)	197.22 ± 70.60	188.35 ± 65.57	205.75 ± 74.26	−3.556	**<0**.**001**
Intraoperative blood transfusion volume (mL)	235.61 ± 167.87	210.72 ± 175.39	259.54 ± 156.94	−3.326	**0**.**001**
Length of hospital stay (days)	12.02 ± 6.12	12.00 ± 6.53	12.05 ± 5.72	−0.696	0.487
Fracture side				0.036	0.849
Left	261 (51.4%)	129 (51.8%)	132 (51.0%)		
Right	247 (48.6%)	120 (48.2%)	127 (49.0%)		
AO/OTA classification				NA	NA
31-A1.1	1 (0.2%)	1 (0.4%)	0 (0.0%)		
31-A1.2	127 (25.0%)	127 (51.0%)	0 (0.0%)		
31-A1.3	121 (23.8%)	121 (48.6%)	0 (0.0%)		
31-A2.2	47 (9.3%)	0 (0.0%)	47 (18.1%)		
31-A2.3	144 (28.3%)	0 (0.0%)	144 (55.6%)		
31-A3.1	8 (1.6%)	0 (0.0%)	8 (3.1%)		
31-A3.2	12 (2.4%)	0 (0.0%)	12 (4.6%)		
31-A3.3	48 (9.4%)	0 (0.0%)	48 (18.5%)		
Reduction quality				9.629	**0**.**002**
Excellent	368 (72.4%)	196 (78.7%)	172 (66.4%)		
Acceptable	140 (27.6%)	53 (21.3%)	87 (33.6%)		
Implant type				2.691	0.260
PFNA	328 (64.6%)	158 (63.5%)	170 (65.6%)		
Gamma 3	71 (14.0%)	41 (16.5%)	30 (11.6%)		
InterTAN	109 (21.5%)	50 (20.1%)	59 (22.8%)		
TAD (mm)	22.97 ± 6.57	22.59 ± 6.53	23.33 ± 6.61	−1.384	0.166
Follow-up time (days)	297.58 ± 174.29	309.76 ± 209.01	285.88 ± 132.00	0.749	0.454

SD, Standard deviation. *n*, Number of cases for each variable.

Continuous variables are expressed as mean ± SD, and categorical variables as *n* (%).

Bold *p* values indicate statistical significance (*p* < 0.05).

### 3.2 Comparison of fixation outcomes between groups

Fixation failure occurred in 16 of 249 patients (6.4%) with complete postoperative lateral-wall status and 17 of 259 (6.6%) with incomplete status (*p* = 0.950). Comparisons of demographic, perioperative, and imaging variables between patients with and without fixation failure are presented in Table 2; no statistically significant between-group differences were detected.

**Table 2:** Comparison of clinical variables between patients with and without fixation failure.

Variable	No fixation failure *n* = 475	Fixation failure *n* = 33	Statistic (z/ χ^2^)	*p*
Age (years)	79.39 ± 10.28	79.52 ± 11.16	−0.303	0.762
BMI (kg/m^2^)	23.10 ± 3.66	23.09 ± 3.64	−0.419	0.675
Sex			2.45	0.118
Male	140 (29.5%)	14 (42.4%)		
Female	335 (70.5%)	19 (57.6%)		
ASA grade			−0.007	0.994
I	10 (2.1%)	1 (3.0%)		
II	255 (53.7%)	17 (51.5%)		
III	202 (42.5%)	15 (45.5%)		
IV	8 (1.7%)	0 (0.0%)		
Time from injury to operation (days)	6.25 ± 3.66	6.00 ± 3.34	−0.150	0.881
Operation time (minutes)	75.98 ± 28.84	72.06 ± 21.30	−0.681	0.496
Intraoperative blood loss (mL)	198.82 ± 72.21	174.24 ± 34.10	−1.843	0.065
Intraoperative blood transfusion volume (mL)	237.31 ± 168.12	211.21 ± 164.77	−0.821	0.411
Length of hospital stay (days)	12.04 ± 6.19	11.79 ± 5.01	−0.311	0.756
Fracture side			1.133	0.287
Left	247 (52.0%)	14 (42.4%)		
Right	228 (48.0%)	19 (57.6%)		
AO/OTA classification			0.438	0.661
31-A1.1	1 (0.2%)	0 (0.0%)		
31-A1.2	119 (25.1%)	8 (24.2%)		
31-A1.3	113 (23.8%)	8 (24.2%)		
31-A2.2	42 (8.8%)	5 (15.2%)		
31-A2.3	134 (28.2%)	10 (30.3%)		
31-A3.1	8 (1.7%)	0 (0.0%)		
31-A3.2	12 (2.5%)	0 (0.0%)		
31-A3.3	46 (9.7%)	2 (6.1%)		
Reduction quality			0.001	0.970
Excellent	344 (72.4%)	24 (72.7%)		
Acceptable	131 (27.6%)	9 (27.3%)		
Implant type			1.125	0.570
PFNA	306 (64.4%)	22 (66.7%)		
Gamma 3	65 (13.7%)	6 (18.2%)		
InterTAN	104 (21.9%)	5 (15.2%)		
Postoperative lateral-wall status			0.004	0.950
Complete	233 (49.1%)	16 (48.5%)		
Incomplete	242 (50.9%)	17 (51.5%)		
TAD (mm)	23.03 ± 6.57	22.09 ± 6.65	−0.889	0.374
Follow-up time (days)	299.18 ± 178.26	274.61 ± 100.12	−0.014	0.989

SD, Standard deviation. *n*, Number of cases for each variable.

Continuous variables are expressed as mean ± SD, and categorical variables as *n* (%).

### 3.3 Survival analysis and Firth logistic regression

The Kaplan–Meier curves (Figure 4) for patients with complete vs. incomplete postoperative lateral-wall status were nearly superimposed, and the log-rank test showed no significant difference in failure-free survival between groups (*p* = 0.778). Cumulative failure rates at 12 and 24 months were comparable between groups, and Cox regression and RMST analyses at 24 months likewise yielded nonsignificant results (age-adjusted Cox *p* = 0.760; RMST *p* = 0.695; Supplementary Table S1).

**Figure 4:**
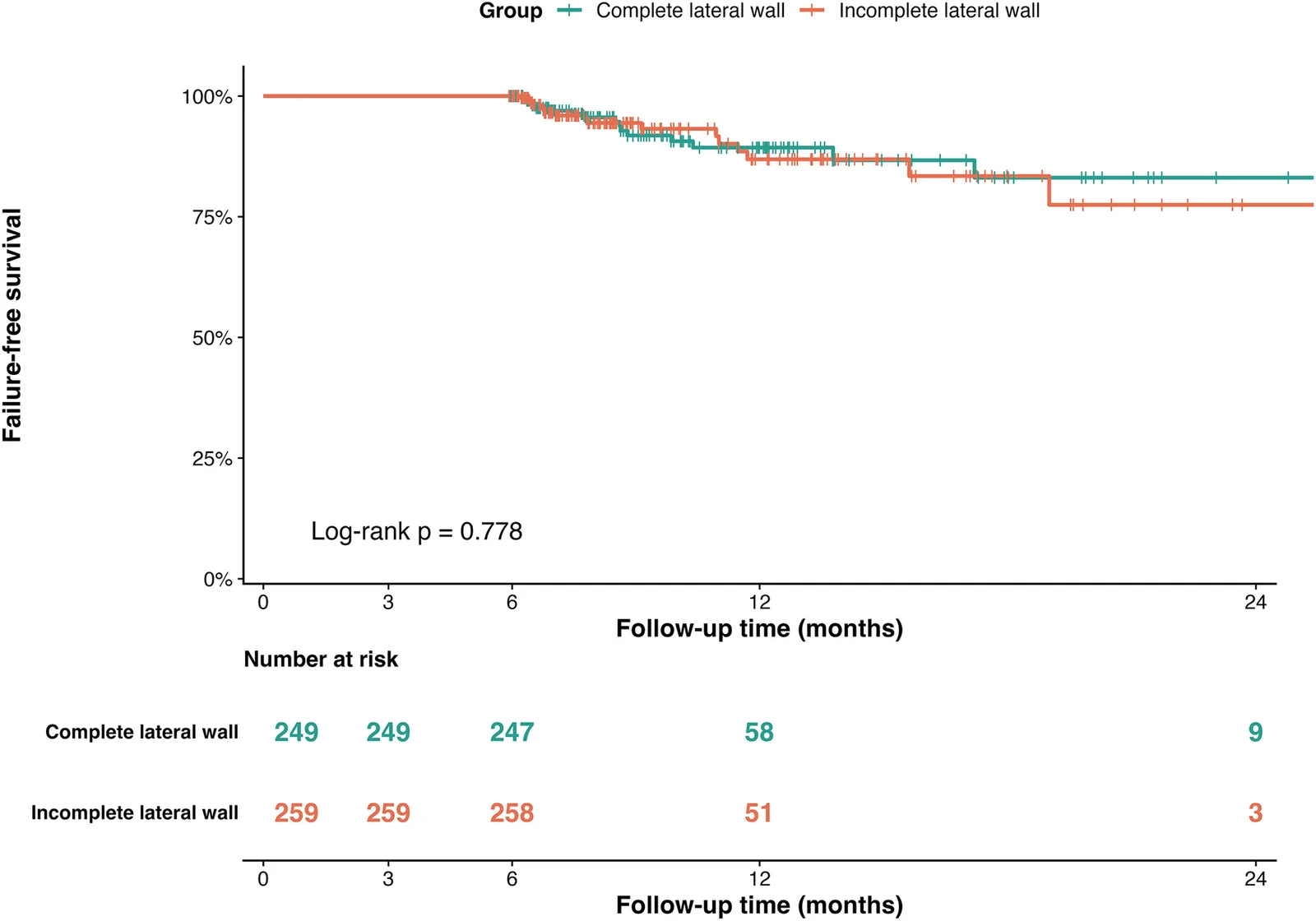
Kaplan–Meier curves for failure-free survival according to postoperative lateral-wall status. The labels “Complete lateral wall” and “Incomplete lateral wall” in the plot denote complete and incomplete postoperative lateral-wall status, respectively. Numbers at risk are shown below the x-axis at 0, 3, 6, 12, and 24 months.

Across the prespecified parsimonious Firth models, no statistically significant association was detected between postoperative lateral-wall status and fixation failure (Table 3). The univariable OR for incomplete vs. complete status was 1.02 (95% CI: 0.51–2.06; *p* = 0.953), and the age- and TAD-adjusted OR in Model A was 1.04 (95% CI: 0.52–2.10; *p* = 0.915). Across Models B–E, adjusted ORs ranged from 0.55 to 1.07, with all 95% CIs including 1. The estimate was similar after additional adjustment for cohort phase (OR: 1.04; 95% CI: 0.52–2.11; *p* = 0.905). The *post hoc* analysis excluding AO/OTA 31-A3 fractures included 440 patients and 31 fixation-failure events and yielded a TAD-adjusted OR of 1.26 (95% CI: 0.61–2.60; *p* = 0.534), broadly consistent with the main findings. No statistically significant associations were detected for TAD or the other clinical covariates, including age, AO/OTA classification, reduction quality, ASA grade, and implant type. Exploratory subgroup and interaction analyses yielded imprecise estimates, with no statistically significant interactions detected. Further details are provided in Supplementary Tables S2, S3 and Figures S3, S4.

**Table 3:** Univariable and multivariable Firth logistic regression analyses for factors associated with fixation failure.

Variable	Univariable OR (95% CI)	*p*	Model A OR (95% CI)^a^	*p*	Model B OR (95% CI)^b^	*p*	Model C OR (95% CI)^c^	*p*	Model D OR (95% CI)^d^	*p*	Model E OR (95% CI)^e^	*p*
Postoperative lateral-wall status	1.02 (0.51–2.06)	0.953	1.04 (0.52–2.10)	0.915	0.55 (0.11–1.82)	0.354	1.04 (0.51–2.11)	0.918	1.04 (0.51–2.10)	0.921	1.07 (0.53–2.16)	0.857
Age	1.00 (0.97–1.04)	0.947	1.00 (0.97–1.04)	0.913	—	—	—	—	—	—	—	—
TAD	0.98 (0.93–1.03)	0.444	0.98 (0.93–1.03)	0.448	0.98 (0.93–1.03)	0.472	0.98 (0.93–1.03)	0.443	0.98 (0.93–1.03)	0.442	0.97 (0.92–1.03)	0.329
AO/OTA classification	1.42 (0.70–2.86)	0.328	—	—	2.29 (0.68–11.79)	0.194	—	—	—	—	—	—
Reduction quality	1.02 (0.45–2.14)	0.968	—	—	—	—	1.03 (0.45–2.19)	0.940	—	—	—	—
ASA grade	1.06 (0.52–2.12)	0.877	—	—	—	—	—	—	1.05 (0.52–2.11)	0.891	—	—
Implant type (Gamma 3 vs PFNA)	1.35 (0.50–3.20)	0.527	—	—	—	—	—	—	—	—	1.29 (0.48–3.08)	0.592
Implant type (InterTAN vs PFNA)	0.72 (0.25–1.75)	0.484	—	—	—	—	—	—	—	—	0.64 (0.21–1.61)	0.354

The odds ratio (OR) for postoperative lateral wall status compares incomplete status with complete status. Data are expressed as ORs with 95% confidence intervals (CIs) obtained from Firth logistic regression. A dash (–) indicates that the variable was not included in the model.

^a^Model A included postoperative lateral-wall status, TAD, and age.

^b^Model B included postoperative lateral-wall status, TAD, and AO/OTA classification.

^c^Model C included postoperative lateral-wall status, TAD, and reduction quality.

^d^Model D included postoperative lateral-wall status, TAD, and ASA grade.

^e^Model E included postoperative lateral-wall status, TAD, and implant type, with PFNA as the reference implant.

## 4 Discussion

Among patients with AO/OTA 31-A extracapsular hip fractures treated with cephalomedullary nailing who achieved satisfactory reduction, we did not detect a statistically significant association between postoperative lateral-wall status and fixation failure. Because patients with poor reduction were excluded by design, these estimates apply to a satisfactorily reduced cohort and do not characterize the overall prognostic relevance of preoperative lateral-wall morphology for treatment difficulty and outcome. Only 33 fixation-failure events occurred, and the age- and TAD-adjusted estimate remained imprecise (OR: 1.04; 95% CI: 0.52–2.10); clinically meaningful associations in either direction therefore cannot be excluded.

Fixation failure and broader postoperative outcomes after cephalomedullary nailing are multifactorial, reflecting fracture morphology and bone quality, reduction quality and cortical support, implant selection and positioning, perioperative events, rehabilitation, and patient vulnerability. Recent observational studies have similarly associated outcomes after fixation of extracapsular hip fractures with reduction quality, infection, postoperative weight-bearing, age, and comorbidity (30, 31). Postoperative lateral-wall status should therefore be interpreted as one component of a broader clinical and mechanical risk profile rather than as an isolated determinant.

### 4.1 Clinical interpretation of postoperative lateral-wall status after cephalomedullary nailing

The lateral wall contributes to proximal femoral stability. However, AO/OTA 31-A3 morphology is conceptually distinct from the 20.5 mm thickness criterion applied to 31-A1 and 31-A2 fractures (14, 17, 28). In the present study, 31-A3 fractures were classified as having incomplete postoperative lateral-wall status on morphological grounds rather than according to the thickness threshold. The *post hoc* sensitivity analysis excluding 31-A3 fractures yielded an imprecise estimate compatible with the primary analysis.

Cephalomedullary nails have a shorter lever arm and may provide an intramedullary buttress, creating a mechanical environment distinct from that of extramedullary constructs (32–35). Consequently, the clinical implications of lateral-wall disruption described for sliding hip-screw fixation may not translate directly to cephalomedullary fixation (14, 17). This distinction provides the rationale for evaluating postoperative lateral-wall status specifically after cephalomedullary nailing.

Prior studies have linked reduction quality and cortical support to fixation outcomes, while reported associations between lateral-wall disruption and outcomes after cephalomedullary nailing have varied (16, 21, 36–38). Because medial cortical support, PACS, and anteromedial cortical contact were not measured as separate study variables, our data cannot determine their relative contributions to fixation failure or compare their prognostic importance with that of postoperative lateral-wall status.

### 4.2 The significance of reduction quality and cortical support

Reduction quality is an established determinant of construct stability and fixation failure after cephalomedullary nailing (2, 16, 39–44). In the present cohort, patients with incomplete postoperative lateral-wall status had a lower proportion of excellent reductions, longer operative times, greater intraoperative blood loss, and greater transfusion volumes. These differences may reflect greater fracture complexity and technical difficulty rather than direct consequences of lateral-wall disruption. One plausible pathway is that adverse fracture morphology makes optimal reduction more difficult, which may in turn compromise construct stability and increase failure risk. Importantly, restricting the cohort to patients with satisfactory reduction conditions the analysis on a postoperative factor that may lie on this pathway. The resulting estimates therefore describe the residual association between postoperative lateral-wall status and fixation failure after satisfactory reduction rather than the overall prognostic relevance of preoperative fracture morphology.

Prior biomechanical and clinical studies support the relevance of medial cortical support and anteromedial cortical contact to load transfer and construct stability, consistent with the PACS reduction concept (22, 24, 25, 45–50). However, medial cortical support, anteromedial cortical contact, and PACS status were not recorded as separate study variables in this cohort, and their influence on the estimated association between postoperative lateral-wall status and fixation failure cannot be determined. Posteromedial fragment morphology, often involving the lesser trochanter, is anatomically distinct from medial cortical support and anteromedial cortical contact and may also influence construct stability (40, 51–55), but it was not separately quantified and its contribution cannot be determined from our data.

### 4.3 Relationship between implant type and fixation failure

The present cohort included PFNA and Gamma 3, which use a single cephalic fixation element, and InterTAN, which uses an integrated dual-screw design. Although biomechanical and comparative studies have suggested potential device-specific advantages, clinical evidence has not demonstrated consistent superiority of one construct across outcomes (56–60).

In the present cohort, fixation failure occurred in 22 of 328 patients treated with PFNA (6.7%), 6 of 71 treated with Gamma 3 (8.5%), and 5 of 109 treated with InterTAN (4.6%). Model E, adjusted for postoperative lateral-wall status and TAD, did not detect statistically significant associations for Gamma 3 vs. PFNA (OR: 1.29; 95% CI: 0.48–3.08; *p* = 0.592) or InterTAN vs. PFNA (OR: 0.64; 95% CI: 0.21–1.61; *p* = 0.354). With only 33 fixation-failure events, including six in the Gamma 3 group and five in the InterTAN group, the analysis was underpowered for reliable implant-specific inference. The confidence intervals remained compatible with clinically meaningful differences and should not be interpreted as evidence of device equivalence. Larger studies with adequate event numbers for each implant are needed to clarify device-specific effects.

### 4.4 Interpretation of TAD as a technical covariate

TAD was retained as a core technical covariate because cephalic-element positioning is an established determinant of cut-out risk (29, 61). In the present analysis, however, TAD was modeled as a single continuous term across PFNA, Gamma 3, and InterTAN and evaluated against the composite fixation-failure endpoint; device-specific or nonlinear associations with individual failure modes were therefore not assessed (62–64). Accordingly, the absence of a statistically significant association should not be interpreted as evidence that TAD is unimportant, and the study was neither designed nor powered to define an optimal TAD threshold across devices.

### 4.5 Limitations

Several limitations warrant consideration. First, only patients with satisfactory reduction were included. The estimates therefore concern the residual association between postoperative lateral-wall status and fixation failure after satisfactory reduction, not the overall prognostic role of preoperative lateral-wall morphology in all surgically treated patients; conditioning on a potential intermediate factor may also have altered the association. Second, AO/OTA 31-A3 fractures were classified as incomplete on morphological grounds rather than by the 20.5 mm criterion applied to 31-A1/A2 fractures. This introduced heterogeneity in the exposure definition, although the sensitivity analysis excluding 31-A3 fractures did not materially change the interpretation. Third, the composite endpoint combined overlapping failure patterns with different mechanisms; small component counts precluded failure-specific analyses. Fourth, PACS, anteromedial cortical contact, medial cortical support, and posteromedial fragment morphology were not measured separately and could not be evaluated as explanatory mechanisms. In addition, 1,007 of 1,420 retrospectively screened patients were excluded for less than 6 months of radiographic follow-up without documented failure during their available follow-up. Records and images were reviewed, but failures after the last contact could not be excluded, and baseline data were unavailable to assess potential selection bias. Only 33 failures occurred. The initial sample-size calculation addressed an unadjusted two-group comparison, and Firth modeling reduced small-sample bias but could not overcome limited precision; the primary 95% CI (0.52–2.10) remained compatible with clinically meaningful associations. Subgroup analyses were exploratory, and the few Gamma 3 and InterTAN failures precluded device-specific inference or equivalence claims. Finally, the single-center, 15-year design remains susceptible to residual temporal confounding and limited generalizability despite cohort-phase adjustment. Although images were independently assessed by two reviewers with senior adjudication, formal reliability statistics were not calculated, and variable follow-up beyond 6 months may have limited detection of late failures.

## 5 Conclusion

Among patients with AO/OTA 31-A extracapsular hip fractures who achieved satisfactory reduction, we did not detect a statistically significant association between postoperative lateral-wall status and fixation failure after cephalomedullary nailing. Because the analysis was restricted to satisfactorily reduced fractures and only 33 failures occurred, a clinically meaningful association cannot be excluded. These findings should not be generalized to patients with poor reduction and do not establish that lateral-wall reconstruction is unnecessary.

## Data Availability

The datasets used and analysed during the current study are available from the corresponding author on reasonable request.
