# Supplementary material for: Postoperative lateral-wall status and fixation failure after cephalomedullary nailing of extracapsular hip fractures: an ambispective cohort study
Source: Front Med (Lausanne). 2026 Oct 28;13:1922104. doi: 10.3389/fmed.2026.1922104 (PMC13751432; doi:10.3389/fmed.2026.1922104)
Supplement: Supplementary file 1 [file Datasheet1.docx]

**Supplementary Information**

**1.Supplementary Methods**

**2.Supplementary Tables**

**3.Supplementary Figures**

**4.References**

**1.Supplementary Methods**

**1.1 Treatment Protocol**

All procedures were performed by three experienced orthopedic trauma surgeons. Throughout the study period, the core operative principles comprised closed reduction with the patient in the supine position, restoration of satisfactory alignment before nail insertion, and adjunctive rod-assisted reduction when closed manipulation alone was insufficient. A Proximal Femoral Nail Antirotation (PFNA), Gamma 3, or InterTAN nail was inserted according to the relevant manufacturer-specific technique; implant availability and selection evolved over time. Perioperative venous thromboembolism prophylaxis was provided according to contemporaneous institutional protocols. Postoperative mobilization followed a criteria-based strategy: partial weight bearing was initiated after radiographic evidence of fracture healing, and full weight bearing was permitted after clinical union.

**1.2 Sample Size Calculation**

Initial sample-size planning was based on an unadjusted comparison of fixation-failure proportions according to postoperative lateral-wall status. Informed by the range of rates reported in previous clinical studies [1, 2], the anticipated proportions were set at 4% among patients with complete postoperative lateral-wall status and 7% among those with incomplete status, corresponding to an expected absolute risk difference of 3 percentage points. Assuming a complete-to-incomplete group ratio of 1:1, an absolute noninferiority margin of 10 percentage points for the risk difference (incomplete minus complete), a one-sided α of 0.025, and 80% power, a normal-approximation calculation for two independent proportions yielded approximately 332 evaluable patients. Allowing for 15% attrition produced a target sample size of approximately 390 patients. This calculation was intended for the unadjusted group comparison and was not a model-based power calculation for the multivariable Firth regression or subgroup analyses.

**1.3 Survival analysis**

Failure-free survival was estimated using Kaplan–Meier (KM) curves, with fixation failure defined as the event. Time to event was calculated from surgery to the first imaging examination confirming fixation failure; patients without fixation failure were censored at the last radiographic follow-up confirming the absence of failure. Follow-up time was expressed in months for visualization, and group differences were assessed using the log-rank test. Cumulative failure rates at 3, 6, 12, and 24 months were estimated as 1 minus the KM failure-free survival probability, with 95% confidence intervals (CIs). These time points were prespecified for analytical reporting and did not represent mandatory visit dates for every patient. Cox proportional hazards models were fitted with postoperative lateral-wall status as the main exposure, and both univariable and age-adjusted models were reported. Proportional hazards assumptions were assessed using Schoenfeld residuals when estimable. Restricted mean survival time (RMST) was calculated using a prespecified truncation time of 24 months, with the between-group difference defined as the incomplete minus complete postoperative lateral-wall status. Hazard ratios and RMST differences were reported with 95% CIs and two-sided p values.

**1.4 Firth’s penalized logistic regression**

Firth penalized logistic regression was used to reduce small-sample bias and improve model stability when evaluating factors associated with fixation failure. The dependent variable was fixation failure during follow-up, and the main exposure was postoperative lateral-wall status, coded as incomplete versus complete. Candidate covariates included age, tip–apex distance (TAD), AO/OTA classification, reduction quality, ASA grade, and implant type. Univariable Firth logistic regression models were first fitted for each candidate predictor. Prespecified parsimonious multivariable models were then constructed rather than a single fully adjusted saturated model. Postoperative lateral-wall status was retained as the main exposure, and TAD was retained as a core technical covariate. Additional covariates were entered one at a time: age in Model A, AO/OTA classification in Model B, reduction quality in Model C, ASA grade in Model D, and implant type in Model E. ORs, 95% CIs, and two-sided p values were reported.

To explore potential temporal confounding across the 15-year study period, a post hoc sensitivity analysis was performed using a parsimonious Firth model that included postoperative lateral-wall status, TAD, and cohort phase (retrospective vs. prospective). Cohort phase represented the transition from retrospective identification between January 2010 and June 2023 to prospective enrollment between July 2023 and October 2025. Because implant type was strongly associated with cohort phase and the number of fixation failure events was limited, implant type and cohort phase were not entered simultaneously into this sensitivity model.

A second post hoc sensitivity analysis excluded AO/OTA 31-A3 fractures and refitted the core Firth model containing postoperative lateral-wall status and TAD. Because 31-A3 fractures were assigned to the incomplete category on the basis of lateral cortical involvement rather than the 20.5-mm thickness threshold, this analysis assessed whether that morphology-based classification influenced the primary estimate.

**1.5 Prespecified subgroup and interaction analyses**

Prespecified subgroup and interaction analyses were performed to explore whether the association between postoperative lateral-wall status and fixation failure differed across clinically relevant strata. postoperative lateral-wall status, coded as incomplete versus complete, was treated as the main exposure. Subgroups were predefined by age (<80 vs. ≥80 years), ASA grade (I–II vs. III–IV), reduction quality (excellent vs. acceptable), and implant type (PFNA, Gamma 3, or InterTAN). Within each subgroup, Firth’s penalized logistic regression was used to estimate the OR for fixation failure associated with incomplete postoperative lateral-wall status. TAD was retained as a core covariate when estimable, and covariates without sufficient within-subgroup variation or estimable coefficients were omitted as needed. Interaction terms between postoperative lateral-wall status and each stratifying factor were further examined in the full cohort. These analyses were considered exploratory.

**1.6 Data Management and Quality Control**

Study procedures followed a standardized quality-control protocol. For the retrospective cohort, a designated investigator screened the institutional surgical database and reviewed the source medical records and relevant images; prospective eligibility was assessed using the same prespecified criteria. For patients with less than 6 months of radiographic follow-up, all available postoperative records and imaging studies through the last documented contact were specifically reviewed before exclusion to identify any fixation failure that had already occurred. Screening results were independently checked by a second investigator, and discrepancies were resolved through joint review of the source data. Lateral-wall thickness, postoperative lateral-wall status, reduction quality, TAD, and fixation outcomes were independently assessed by two trained orthopedic reviewers using prespecified definitions. Baseline and immediate postoperative images were assessed without reference to subsequent fixation outcomes, whereas follow-up images were reviewed without access to the recorded postoperative lateral-wall status or other predictor data. Disagreements were adjudicated by a third senior orthopedic trauma surgeon. The same assessment procedures were applied in both cohort phases. Data were entered by a designated team member and independently audited by another; any queries were resolved against the original medical records, case report forms, and electronic files.

Missingness was evaluated using Little’s MCAR test as a diagnostic. Multiple imputation by chained equations was conducted under a missing-at-random working assumption using the mice package in R. Twenty imputed datasets were generated using predictive mean matching with five donors and 20 iterations per chain. The imputation model included sex, age, BMI, ASA grade, time from injury to operation, operation time, intraoperative blood loss, intraoperative blood transfusion volume, length of hospital stay, fracture side, AO/OTA classification, reduction quality, implant type, TAD, follow-up time, postoperative lateral-wall status, fixation failure status, and cohort phase; patient ID was excluded from the predictor matrix. Convergence was assessed using trace plots and comparisons of observed and imputed distributions. Models involving imputed covariates were fitted separately in each completed dataset, and regression coefficients and their variances were combined using Rubin’s rules.

**2.Supplementary Tables**

**Table S1 Cumulative failure rates, hazard ratios, and RMST difference between complete and incomplete postoperative lateral-wall status groups**

| Time point (months) | Cumulative failure – complete, % (95% CI) | Cumulative failure – incomplete, % (95% CI) | HR (unadjusted) (95% CI) | HR (adjusted*) (95% CI) | RMST difference (days) (95% CI) |
| --- | --- | --- | --- | --- | --- |
| 3 | 0.0 (0.0–0.0) | 0.0 (0.0–0.0) |  |  |  |
| 6 | 0.0 (0.0–0.0) | 0.0 (0.0–0.0) |  |  |  |
| 12 | 10.7 (4.8–16.2) | 13.1 (5.7–19.9) |  |  |  |
| 24 | 16.9 (6.3–26.3) | 22.5 (6.8–35.6) | 1.10 (0.56–2.18), p=0.778 | 1.11 (0.56–2.20), p=0.760 | -9.9  (-59.7–39.8), p=0.695 |
| *Adjusted for age. | | | | | |

**Table S2 Results of prespecified subgroup analyses**

| Model | Variable | OR | 95% CI | p-value |
| --- | --- | --- | --- | --- |
| Age group: <80 | Group (incomplete vs complete) | 0.94 | (0.28–3.06) | 0.914 |
| Age group: ≥80 | Group (incomplete vs complete) | 1.06 | (0.45–2.55) | 0.888 |
| ASA group: ASA I–II | Group (incomplete vs complete) | 1.33 | (0.52–3.47) | 0.552 |
| ASA group: ASA III–IV | Group (incomplete vs complete) | 0.76 | (0.27–2.15) | 0.604 |
| Reduction: Excellent | Group (incomplete vs complete) | 0.69 | (0.29–1.57) | 0.383 |
| Reduction: Acceptable | Group (incomplete vs complete) | 3.73 | (0.81–35.76) | 0.097 |
| Implant: PFNA | Group (incomplete vs complete) | 0.93 | (0.39–2.21) | 0.875 |
| Implant: Gamma 3 | Group (incomplete vs complete) | 2.47 | (0.50–15.06) | 0.265 |
| Implant: InterTAN | Group (incomplete vs complete) | 0.61 | (0.10–3.25) | 0.555 |
| *Subgroup and interaction analyses were exploratory. Models were fitted within each subgroup using Firth logistic regression with postoperative lateral-wall status as the main exposure; covariates without within-subgroup variation or without estimable coefficients were omitted automatically. 95% CIs were rounded to two decimal places. | | | | |

**Table S3. Distribution of key variables by cohort phase and results of post hoc sensitivity analyses**

| **Panel A. Distribution by cohort phase** | | | | |
| --- | --- | --- | --- | --- |
| **Variable** | **Category** | **Retrospective cohort (n=365), n (%)** | **Prospective cohort (n=143), n (%)** | **p-value** |
| Postoperative lateral-wall status | Complete | 179 (49.0) | 70 (49.0) | 1 |
|  | Incomplete | 186 (51.0) | 73 (51.0) |  |
| Implant type | PFNA | 297 (81.4) | 31 (21.7) | <0.001 |
|  | Gamma 3 | 63 (17.3) | 8 (5.6) |  |
|  | InterTAN | 5 (1.4) | 104 (72.7) |  |
| Fixation outcome | Fixation failure | 27 (7.4) | 6 (4.2) | 0.232 |
| **Panel B. Post hoc cohort-phase-adjusted Firth logistic regression** | | | | |
| **Model** | **Variable** | **OR** | **95% CI** | **p-value** |
| Cohort-phase-adjusted model | Postoperative lateral-wall status (incomplete vs complete) | 1.04 | (0.52–2.11) | 0.905 |
|  | TAD (per mm) | 0.98 | (0.92–1.03) | 0.414 |
|  | Cohort phase (prospective vs retrospective) | 0.58 | (0.22–1.32) | 0.204 |
| **Panel C. Sensitivity analysis excluding AO/OTA 31-A3 fractures** | | | | |
| **Model** | **Variable** | **OR** | **95% CI** | **p-value** |
| A3-excluded core model | Postoperative lateral-wall status (incomplete vs complete) | 1.26 | (0.61–2.60) | 0.534 |
|  | TAD (per mm) | 0.98 | (0.92–1.03) | 0.416 |
| *Panel A was descriptive. Categorical data are presented as n (%). Postoperative lateral-wall status and fixation-failure proportions were compared between cohort phases using Fisher’s exact test, and implant distribution was compared using Pearson’s chi-square test.  *Panel B was fitted in the full cohort using Firth logistic regression, with postoperative lateral-wall status as the main exposure and TAD and cohort phase as covariates (N = 508; 33 fixation-failure events). Implant type and cohort phase were not entered simultaneously because implant use differed markedly by cohort phase and the number of events was limited.  *Panel C excluded AO/OTA 31-A3 fractures and refitted the core Firth model containing postoperative lateral-wall status and TAD (N = 440; 31 fixation-failure events). ORs are presented with profile-likelihood 95% CIs and rounded to two decimal places. | | | | |

**3. Supplementary Figures**

**(Figure S1) Representative assessment of fracture reduction quality.** Reduction quality was classified according to the Baumgaertner–Solberg criteria. The alignment criterion required a normal or slightly valgus neck-shaft angle on the anteroposterior radiograph and <20° of angulation on the lateral radiograph; the displacement criterion required <4 mm of displacement on both projections. An excellent reduction met both criteria, an acceptable reduction met either criterion, and a poor reduction met neither. **Figure S1A** and **Figure S1B** show acceptable and excellent reductions, respectively.

**(Figure S2) Measurement of TAD.** TAD was calculated as follows: TAD = (Xap × Dtrue/Dap) + (Xlat × Dtrue/Dlat), where Xap and Xlat represent the distances from the tip of the cephalic fixation element to the femoral head apex on the anteroposterior and lateral radiographs, respectively; Dtrue is the actual implant diameter, and Dap and Dlat are its measured radiographic diameters on the corresponding projections.

**(Figure S3) Forest plots of Firth’s logistic regression analysis. Figure S3A** displays univariable associations between candidate predictors and fixation failure. **Figure S3B** displays adjusted estimates for incomplete versus complete postoperative lateral-wall status across the prespecified parsimonious multivariable models (Models A–E). ORs with 95% CIs are shown. Within the plotted figures, “postoperative lateral-wall status” denotes postoperative lateral-wall status as defined in this study, and “fixation failure” denotes the prespecified fixation-failure endpoint.

**(Figure S4) Forest plot of prespecified subgroup analyses.** Firth’s penalized logistic regression was used to estimate the association between incomplete versus complete postoperative lateral-wall status and fixation failure within strata defined by age, ASA grade, reduction quality, and implant type. The “Complete” and “Incomplete” groups in the plot denote complete and incomplete postoperative lateral-wall status, respectively. TAD was retained as a core covariate when estimable. ORs with 95% CIs are shown, and p values for interaction are displayed where applicable; analyses were exploratory.

4. **References**

[1].	Fan J, Xu X, Zhou F, Zhang Z, Tian Y, Ji H, et al. Risk factors for implant failure of intertrochanteric fractures with lateral femoral wall fracture after intramedullary nail fixation. Injury. 2021;52(11):3397-403.

[2].	Tang Y, Wang D, Wang L, Xiong W, Fang Q, Lin W, et al. The PFNA in treatment of intertrochanteric fractures with or without lateral wall fracture in elderly patients: a retrospective cohort study. Eur J Med Res. 2023;28(1):380.
